# A Rondelay (Without Cadenza) By The Virion Of Influenza

**DOI:** 10.3201/eid1402.079999

**Published:** 2008-02

**Authors:** Edwin D. Kilbourne

Now you have me―sucrose-banded,
Enveloped and negative-stranded
Spiked and cleaved and slightly dented
Into pieces eight, segmented―
Without mercy I’ve been strained
Through filters―then been bromelained, and
Torn apart by each detergent
With a haste unseemly, urgent―
All my helices displayed
Just to show you how I’m made.
My polypeptides have been mapped
My hemagglutinin unwrapped―
All my sequences are clear―
All the way from Arg to Ser.
With techniques sharp and newly honed
My very genes have now been cloned―
Transplanted to an alien host
Wherein my evanescent ghost
As solitary as an elf
Has managed to express myself.
And scientists have labored nights
To probe my antigenic sites
Some fresh from school with new diplomas
(Aided by their hybridomas)
Scramble up trimeric slopes
Counting all my epitopes
And every Ph.D. or pupil
Utterly devoid of scruple
Mates me with complete abandon―
Asks, then, why my genes are random
Can I kiss and never tell
When genotyped upon a gel?
Which, if inspected with acuity
Will document my promiscuity―
Must you write, in fat reports
How flagrantly I reassort?
No boundaries have my misbehavin’
Horsey set or duck or avian―
In other moments less sublime
You’ve put my perils before swine!
And yet in 1981
Despite the work that has been done
The epidemics come and go
As regular as winter snow.
And people cough and people die
And all of you still wonder why.
I’m so perverse and ever mutable
And so eternally unscrutable.
But think about just what you’d do
If there were really
No more flu!

